# First-line single-agent chemotherapy for patients with recurrent or metastatic gastric cancer with poor performance status

**DOI:** 10.3892/etm.2012.644

**Published:** 2012-07-23

**Authors:** JUN-EUL HWANG, HA-NA KIM, DAE-EUN KIM, HYUN-JEONG SHIM, WOO-KYUN BAE, EU-CHANG HWANG, SANG-HEE CHO, IK-JOO CHUNG

**Affiliations:** 1 Division of Hematology-Oncology, Department of Internal Medicine and; 2 Department of Urology, Chonnam National University Hwasun Hospital, Jeonnam, Republic of Korea

**Keywords:** chemotherapy, stomach neoplasms, recurrence, metastasis

## Abstract

Combination chemotherapy is a standard treatment approach in advanced gastric cancer. However, combination chemotherapy for advanced gastric cancer is often associated with severe treatment-related toxicities and most oncologists are reluctant to perform combination chemotherapy in patients with a poor clinical condition. We retrospectively investigated the efficacy and tolerability of single-agent chemotherapy in patients with recurrent or metastatic gastric cancer with poor performance status (PS). We reviewed advanced gastric adenocarcinoma patients who received first-line single-agent palliative chemotherapy due to poor PS between June 2006 and December 2010. A total of 125 patients with Eastern Cooperative Oncology Group (ECOG) PS 2–3, whose general condition did not allow combination chemotherapy, were enrolled. Four single agents were used: TS-1 (n=63), paclitaxel (n=42), irinotecan (n=15) and capecitabine (n=5). The median age was 66 years, with a range of 25–81 years. The percent response rate and rate of stable disease (SD) were 19.2 and 35.2%, respectively, giving a disease control rate of 54.4%. The median progression-free survival (PFS) was 3.9 months (95% CI, 2.73–5.06). The median overall survival (OS) was 9.1 months (95% CI, 7.70–10.56) with a 1-year survival rate of 31.2%. Multivariate analysis demonstrated that the independent prognostic factors for OS were chemotherapy regimen (capecitabine) [reference: TS-1, hazard ratio (HR), 5.00; 95% CI, 1.81–13.81; P=0.002], no second-line chemotherapy (HR, 2.3; 95% CI, 1.48–3.57; P=0.001), bone metastasis (HR, 2.73; 95% CI, 1.22–6.09; P=0.014), ECOG PS 3 (HR, 38.10; 95% CI, 13.72–105.78; P=0.001), Glasgow prognostic score (GPS) ≥1 (HR, 1.88; 95% CI, 1.24–2.85; P=0.003) and chemotherapy response [SD + progressive disease (PD) + not evaluable (NE); HR, 2.37; 95% CI, 1.39–4.05; P=0.002)]. First-line single-agent palliative chemotherapy demonstrated a relatively good clinical efficacy for recurrent or metastatic gastric cancer patients with poor PS.

## Introduction

Recurrent or metastatic gastric cancer has a poor prognosis but chemotherapy improves survival and possibly provides significant palliation of symptoms. While newer agents for advanced gastric cancer have been developed in recent years, including TS-1, capecitabine, taxanes, oxaliplatin and irinotecan, the prognosis of unresectable, recurrent or metastatic gastric cancer patients remains extremely poor, with a survival of only 6–13 months (1–3).

Eastern Cooperative Oncology Group performance status (ECOG PS) is the most important parameter for predicting response to chemotherapy and survival, and is associated with a number of factors, including age, comorbidity and nutritional status (4,5). ECOG PS 2 had a significant negative impact on survival in a number of other studies. PS classifications of 0–1 and 2–3 are generally used to stratify patients in phase III trials on advanced gastric cancer due to their well-known impact on survival (6–8).

In recurrent or metastatic gastric cancer, patients are likely to suffer from poor general condition due to anorexia and weight loss, often as a consequence of peritoneal carcinomatosis. These patients have been excluded from clinical trials and chemotherapeutic options are limited (9,10). Most oncologists select cisplatin-based combination chemotherapy for recurrent or metastatic gastric cancer patients with good PS in the first-line setting. Multidrug combination chemotherapy regimens have generally provided higher response rates, but also more substantial toxicities (11) and do not significantly improve overall survival (OS). These toxicities reduce their value as a palliative treatment and are of particular concern in patients whose PS is compromised (12). Selecting a chemotherapy regimen for an individual patient is a common clinical situation and factors such as the extent of disease and potential toxicities must be considered, especially for patients with poor PS.

The single-agent chemotherapy drug TS-1 (Taiho Pharmaceutical Company, Tokyo, Japan) has demonstrated good clinical efficacy for advanced gastric cancer (13). Therefore, in the present study, we retrospectively evaluated patients with unresectable, recurrent or metastatic gastric cancer who received first-line single-agent palliative chemotherapy due to poor PS (ECOG PS 2–3).

## Patients and methods

### Patients

We evaluated patients with advanced gastric cancer who had received first-line single-agent palliative chemotherapy between June 2006 and December 2010 at Chonnam National University Hwasun Hospital (Gwangju, Korea). Patients were staged using a combination of endoscopy, computed tomography (CT) scans of the chest and abdomen and positron emission tomography or bone scans when clinically indicated.

The criteria for case inclusion were as follows: i) histologically confirmed gastric adenocarcinoma; ii) no prior chemotherapy or radiotherapy, with the exception of adjuvant treatment; iii) presence of metastatic disease; and iv) availability of clinical data at the initiation of therapy and during follow-up. Of the 201 patients screened, 125 fulfilled the inclusion criteria and were enrolled in this retrospective analysis. We collected follow-up patient data from the cancer registry. All data were prospectively recorded and only the survival data were updated at the time of analyses.

ECOG PS was evaluated according to the ECOG criteria. The clinical tumor response was assessed radiologically by CT scanning after every 2 or 3 courses of chemotherapy according to the Response Evaluation Criteria in Solid Tumors (RECIST version 1.0) (14).

The chemotherapy regimens included TS-1 (n=63), paclitaxel (n=42), irinotecan (n=15) and capecitabine (n=5). TS-1 was given at a dose of 80 mg/m^2^/day for 4 weeks, followed by a 2-week rest (13). Paclitaxel was administered at a dose of 80 mg/m^2^ by intravenous infusion, weekly for 3 weeks of a 4 week cycle (days 1, 8 and 15 of each cycle) (15). Irinotecan was administered at a dose of 150 mg/m^2^ by intravenous infusion every 2 weeks (days 1 and 15 of each cycle) (16,17). Capecitabine was administered at a dose of 1,250 mg/m^2^/day for 2 weeks, followed by a 1-week rest (18).

Treatment was continued until the occurrence of disease progression, lack of clinical benefit, unacceptable toxicity or patient refusal. Toxicities were graded according to the National Cancer Institute Common Terminology Criteria for Adverse Events version 3.0 (CTCAE). The dose of the subsequent cycles was adjusted according to the toxic effects that developed during the preceding cycle.

This study was approved by the Institutional Review Board of Chonnam National University Medical School Research Institution.

### Statistics

Kaplan-Meier analysis was applied to assess factors affecting OS and progression-free survival (PFS) and the significance of differences between survival curves was determined by the log-rank test. OS was defined as the period from the date of the first course of chemotherapy to the date of mortality from any cause. PFS was defined as the period from the date of the first course of chemotherapy to the date of disease progression or mortality, whichever occurred first. If neither event had occurred at the time of the last record, the patient was censored at that time. The factors included in univariate survival analysis were age, gender, histological grade, Lauren classification, previous gastrectomy, chemotherapy regimen, chemotherapy response, second-line chemotherapy, liver metastasis, peritoneal metastasis, bone metastasis, albumin, C-reactive protein (CRP) concentration and Glasgow prognostic score (GPS). GPS is known to be associated with prognosis in gastric cancer. Patients with both an elevated CRP concentration (>1.0 mg/dl) and hypoalbuminemia (<3.5 mg/dl) were assigned a GPS of 2. Patients in whom only 1 of these biochemical abnormalities was present were assigned a GPS of 1 and patients with normal CRP and albumin levels were assigned a score of 0 (19). Multivariate regression analysis using the Cox proportional hazards regression model (the stepwise forward procedure), was performed to achieve an adjusted hazard ratio (HR) to determine prognostic factors for OS and PFS. A two-tailed P<0.05 was considered to indicate a statistically significant result for all analyses. The SPSS software package, version 17.0 (SPSS, Inc., Chicago, IL, USA) was used for statistical analysis.

## Results

### Patient characteristics

The baseline characteristics of the 125 patients are listed in Table I. The median follow-up time was 9.1 months, with a range of 1.7–34.2 months. The median age of the patients was 66 years, with a range of 25–81 years. A total of 90 patients (72%) were male and 110 patients (88%) had a ECOG PS of 2. A total of 97 patients (77.6%) had measurable metastatic lesions. A total of 42 patients (33.6%) had liver metastases and 48 patients (38.4%) and 7 patients (5.6%) had peritoneal and bone metastases, respectively. A total of 61 patients (48.8%) had undergone gastrectomy prior to palliative chemotherapy. A total of 68 patients (54.4%) had an elevated CRP level (>1 mg/dl) and 38 patients (30.4%) had hypoalbuminemia (<3.5 mg/dl). A total of 65 patients (52%) received second-line chemotherapy.

### Toxicities

A total of 482 chemotherapy cycles were administered. The patients received a median of 3 (range, 1–12) cycles. The most common reason for treatment discontinuation was disease progression. Toxicity was assessed in all patients. The chemotherapy toxicities during the total treatment courses are summarized in Table II. Hematological toxicities were relatively common. The incidence of grade 3 or 4 leukopenia and neutropenia were 18 and 14%, respectively. Four patients developed febrile neutropenia. Three of these patients recovered with systemic antibiotics and granulocyte colony-stimulating factor, while 1 patient succumbed to neutropenic sepsis after receiving weekly paclitaxel. Anemia was the most common adverse event, since 79 of the 125 patients (63.2%) had anemia before the initiation of the chemotherapy. Three patients (2%) had grade 3 thrombocytopenia; 2 in the paclitaxel group and 1 in the irinotecan group. Non-hematological toxicities consisted of asthenia/anorexia (21%), nausea/vomiting (18%), mucositis (17%), diarrhea (23%) and hand-foot syndrome (6%). Peripheral neuropathy developed in 21 patients (17%) but was mild with the exception of 1 patient. All patients with peripheral neuropathy were in the paclitaxel group.

### Response and survival

The most commonly used first-line single-agent chemotherapy regimen was TS-1 (n=63, 50.4%). The specific chemotherapy regimens are shown in Table III. Of a total of 125 patients, 6 could not be evaluated for responses because of early discontinuation of therapy. A complete response (CR) to chemotherapy was achieved in 3 patients (2.4%) and a partial response (PR) in 21 patients (16.8%). The percentage of stable disease (SD) was 35.2%, giving an overall response rate of 19.2% and a disease control rate of 54.4%. Objective tumor response to each regimen was 19% for TS-1, 21.4% for paclitaxel, 13.3% for irinotecan and 20% for capecitabine. The chemotherapy results are shown in Table IV.

**Figure 1:**
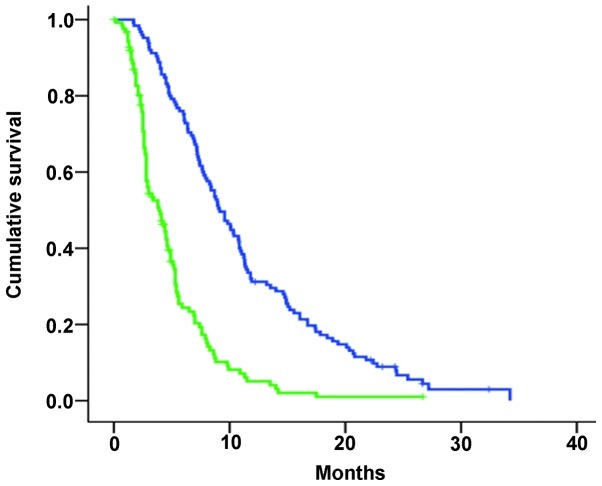
OS and PFS curve for all patients. The median OS and PFS were 9.1 (95% CI, 7.70–10.56) and 3.9 months (95% CI, 2.73–5.06), respectively, with a 1-year survival rate of 31.2% (blue line, OS; green line, PFS. OS, overall survival; PFS, progression-free survival.

The median PFS was 3.9 months (95% CI, 2.73–5.06). The median OS was 9.1 months (95% CI, 7.70–10.56) with a 1-year survival rate of 31.2% (Fig. 1). In univariate analysis, the median OS was significantly shorter for patients with the following clinical factors: no second-line chemotherapy, response of stable and progressive disease (PD) to chemotherapy, ECOG PS 3, CRP >1.0 mg/dl and GPS ≥1 (Table V). Univariate analysis also demonstrated that 3 clinical factors were significantly associated with a shorter PFS; these factors included response of SD and PD to chemotherapy, ECOG PS 3 and GPS ≥1 (Table VI).

Multivariate regression analysis using the Cox proportional hazards regression model (the stepwise forward procedure) was performed. The result of the analysis identified the independent poor prognostic factors for OS and PFS (Table VII). The independent poor prognostic factors for OS were chemotherapy regimen (capecitabine; reference: TS-1, HR, 5.00; 95% CI, 1.81–13.81; P=0.002), no second-line chemotherapy (HR, 2.30; 95% CI, 1.48–3.57; P=0.001), bone metastasis (HR, 2.73; 95% CI, 1.22–6.09; P=0.014), ECOG PS 3 (HR, 38.10; 95% CI, 13.72–105.78; P=0.001), GPS ≥1 (HR, 1.88; 95% CI, 1.24–2.85; P=0.003) and chemotherapy response [SD + PD + not evaluable (NE); HR, 2.37; 95% CI, 1.39–4.05; P=0.002)]. The independent prognostic factors for PFS were chemotherapy regimen (capecitabine; reference: TS-1; HR, 4.20; 95% CI, 1.64–10.85; P=0.003), ECOG PS 3 (HR, 5.86; 95% CI, 3.07–11.20; P=0.001) and chemotherapy response (SD + PD + NE; HR, 2.41; 95% CI, 1.44–4.04; P=0.001). After first-line failure, second-line chemotherapy was administered in 65 patients (52%). OS was longer in the group able to receive second-line chemotherapy (11.2 vs. 6.6 months, P=0.004).

## Discussion

Currently, fluoropyrimidine and cisplatin combination chemotherapy is accepted as a standard regimen by numerous oncologists. However, cisplatin is associated with significant toxicity and usually requires careful clinical monitoring and supportive care, including intensive intravenous hydration. Patients with poor PS usually have several comorbidities and are also more vulnerable to the toxicity of chemotherapy (11). Therefore, we evaluated the efficacy of single-agent chemotherapy with TS-1, paclitaxel, capecitabine and irinotecan. TS-1, paclitaxel and capecitabine are the counterparts of a cisplatin-based combination chemotherapy for advanced gastric cancer (11).

PS is an indicator of a patient’s global ability and it correlates with survival time. Preoperative ECOG PS 2–3 in incurable gastric cancer patients is associated with a 1-year survival rate of 17%, compared with 43% for ECOG PS 0–1 patients (20). A phase II study of TS-1 demonstrated modest activity of TS-1 in gastric cancer patients with poor PS. The response rate was 12%, with a 1-year survival rate of 29%. This study included a more heterogeneous group of patients who received TS-1 as first- or second-line treatment (12). In a prospective trial of TS-1 plus cisplatin versus TS-1 alone (SPIRITS trial), the median PFS and OS of the TS-1 group were 4.0 and 11.0 months, respectively, with a 1-year survival rate of 46.7% and almost all patients (97%) in this trial had a ECOG PS 0–1 (2). Our study demonstrated median PFS and OS of 3.9 and 9.1 months, respectively, with a 1-year survival rate of 31.2% and a response rate of 19.2%. This suggests a relatively good efficacy of single-agent chemotherapy in gastric cancer patients in this poor PS population.

In the present study, there were no statistically significant differences between the regimens used, with the exception of capecitabine. TS-1 is an oral anticancer drug that combines tegafur, a prodrug of fluouracil, with 5-chloro-2,4-dihydropyrimidine (CDHP) and potassium oxonate (21). TS-1 is widely used as first-line treatment for advanced gastric cancer in Asian countries. Phase II studies of TS-1 have noted responses of 44–54% in patients with advanced gastric cancer, but TS-1 has lower responses of 13–33% in this type of cancer when combined with poor clinical condition, including advanced age and poor PS (12,22,23). Weekly paclitaxel and irinotecan regimens are usually used as second-line treatment for advanced gastric cancer with a response rate of 14–21% (24,25). Although direct comparison is difficult, our results are comparable to others for single-agent chemotherapy regimens in poor performers or pretreated patients. Capecitabine is mostly used as a combination regimen and when used as a single-agent, it demonstrates a response rate of 26–34% (18). In our study, capecitabine demonstrated a relatively low efficacy in PFS and OS, but we propose that this was due to the small number of patients in the capecitabine group.

We also evaluated the clinical factors associated with poor survival in this subgroup of recurrent or metastatic gastric cancer. No second-line chemotherapy, bone metastasis, ECOG PS 3, responses to chemotherapy (SD + PD + NE) and GPS ≥1 were significant survival predictors. It has been reported that GPS is associated with prognosis in various types of cancer, including non-small cell lung, gastric, colorectal, pancreatic and breast cancers. Previously, we reported that GPS is a useful predictor of survival in patients with recurrent or metastatic gastric cancer receiving palliative chemotherapy (19). GPS was a useful prognostic indicator in this subgroup of recurrent or metastatic gastric cancer.

In Korea, it has been common practice for patients who fail first-line palliative chemotherapy to receive second-line chemotherapy and Kang *et al* reported that salvage chemotherapy (second- or third-line chemotherapy) in advanced gastric cancer resulted in significant prolongation of survival when compared with best supportive care (17). Second-line chemotherapy was also a significant survival predictor in our study of recurrent or metastatic gastric cancer patients with poor performance status.

In conclusion, first-line single-agent palliative chemotherapy is tolerated and has a relatively good clinical efficacy for the treatment of recurrent or metastatic gastric cancer patients with poor PS.

## Figures and Tables

**Table I.** Patient characteristics.

Characteristics	No. of patients (n=125)
Age (years)	
Median (range)	66 (25–81)
Gender	
Male/female	90/35
ECOG PS	
2/3	110/15
Histological grade	
Adenocarcinoma, WD	10
Adenocarcinoma, MD	38
Adenocarcinoma, PD	57
Signet ring cell carcinoma	12
Unknown	8
Lauren classification	
Diffuse	40
Intestinal	63
Mixed	14
Unknown	8
Previous gastrectomy (yes/no)	61/64
Second-line chemotherapy (yes/no)	65/60
Metastatic site	
Liver	42
Peritoneum	48
Bone	7
Albumin (g/dl)	
<3.5	38
≥3.5	87
CRP (mg/dl)	
≤1.0	57
>1.0	68
GPS	
0/1/2	50/44/31

ECOG PS, Eastern Cooperative Oncology Group performance status; WD, well-differentiated; MD, moderately differentiated; PD, poorly differentiated; CRP, C-reactive protein; GPS, Glasgow prognostic score.

**Table II:** Toxicities.

	No. of patients (n=125)					
Toxicity	Grade 1	Grade 2	Grade 3	Grade 4	All grades (%)	Grade 3/4 (%)
Hematological						
Leukopenia	34	14	14	9	57	18
Neutropenia (febrile)	33	13	11 (3)	6 (1)	50	14
Anemia	45	47	6	4	82	8
Thrombocytopenia	13	9	3	0	20	2
Non-hematological						
Asthenia/anorexia	9	4	13	0	21	10
Nausea/vomiting	8	5	10	0	18	8
Mucositis	13	8	0	0	17	0
Diarrhea	16	5	8	0	23	6
Peripheral neuropathy	9	11	1	0	17	1
Hand-foot syndrome	3	3	1	0	6	1

**Table III:** Chemotherapy regimens (n=125).

Regimen	No. of patients	%
TS-1	63	50.4
Paclitaxel	42	33.6
Irinotecan	15	12
Capecitabine	5	4

**Table IV:** Response to chemotherapy (n=125).

Response	No. of patients	%
Complete response	3	2.4
Partial response	21	16.8
Stable disease	44	35.2
Progressive disease	51	40.8
Not evaluable	6	4.8

**Table V:** Univariate analysis of clinical factors for overall survival (n=125).

	mOS months (95% CI)	P-value
Age (years)		
<66	8.7 (6.29–11.1)	0.506
≥66	9.5 (7.64–11.49)	
Gender		
Male	8.7 (7.09–10.30)	0.552
Female	10.8 (8.5–13.1)	
Type		
Diffuse	8.9 (7.89–10.04)	0.433
Intestinal	10.1 (7.61–12.58)	
Mixed	8.3 (0.00–17.10)	
Unknown	7.0 (2.98–11.01)	
Previous gastrectomy		
Yes	9.5 (7.85–11.27)	0.226
No	8.4 (5.78–11.01)	
Liver metastasis		
Yes	7.2 (4.34–10.05)	0.355
No	9.5 (8.21–10.92)	
Peritoneal metastasis		
Yes	8.9 (7.27–10.59)	0.567
No	9.5 (7.56–11.57)	
Chemotherapy regimen		
TS-1	9.5 (7.27–11.86)	0.830
Paclitaxel	9.0 (6.91–11.15)	
Irinotecan	8.2 (5.57–10.96)	
Capecitabine	6.3 (0.00–13.45)	
Second-line chemotherapy		
Yes	11.2 (10.51–12.02)	0.004
No	6.6 (5.57–7.76)	
Bone metastasis		
Yes	7.3 (7.04–7.55)	0.390
No	9.5 (8.08–11.04)	
Chemotherapy response		
CR+PR	14.8 (10.13–19.60)	0.001
SD+PD	8.7 (7.30–10.09)	
NE	3.0 (0.00–7.66)	
Histological grade		0.379
Adenocarcinoma, WD	7.2 (4.77–9.62)	
Adenocarcinoma, MD	10.7 (8.19–13.33)	
Adenocarcinoma, PD	8.9 (7.06–10.86)	
Signet ring cell carcinoma	13.5 (5.89–21.17)	
Unknown	7.0 (2.98–11.01)	
ECOG PS		
2	10.3 (8.91–11.75)	0.001
3	3.0 (2.36–3.63)	
Albumin (g/dl)		
<3.5	7.4 (6.59–8.20)	0.055
≥3.5	10.3 (8.50–12.16)	
CRP (mg/dl)		
≤1.0	11.8 (8.26–15.33)	0.001
>1.0	7.1 (6.15–8.11)	
GPS		
0	11.8 (7.99–15.60)	0.001
≥1	7.4 (6.39–8.40)	

mOS, median overall survival; CR, complete response; PR, partial response; SD, stable disease; PD, progressive disease; NE, not evaluable; WD, well-differentiated; MD, moderately differentiated; PD, poorly differentiated; ECOG PS, Eastern Cooperative Oncology Group performance status; CRP, C-reactive protein; GPS, Glasgow prognostic score.

**Table VI:** Univariate analysis of clinical factors for progression-free survival (n=125).

	mPFS months (95% CI)	P-value
Age (years)		
<66	3.2 (2.23–4.30)	0.576
≥66	4.3 (3.38–5.49)	
Gender		
Male	3.9 (2.66–5.27)	0.286
Female	3.7 (2.00–5.52)	
Type		
Diffuse	4.1 (1.90–6.29)	0.853
Intestinal	3.9 (2.73–5.20)	
Mixed	2.9 (0.00–5.86)	
Unknown	2.8 (2.06–3.60)	
Previous gastrectomy		
Yes	3.9 (2.34–5.59)	0.704
No	3.8 (2.11–5.61)	
Liver metastasis		
Yes	2.8 (2.59–3.07)	0.570
No	4.3 (3.59–5.13)	
Peritoneal metastasis		
Yes	2.8 (1.48–4.11)	0.377
No	4.3 (3.30–5.42)	
Chemotherapy regimen		
TS-1	4.3 (3.58–5.15)	0.133
Paclitaxel	3.7 (2.93–4.60)	
Irinotecan	2.8 (1.82–3.84)	
Capecitabine	2.0 (1.56–2.56)	
Second-line chemotherapy		
Yes	3.9 (2.44–5.48)	0.933
No	3.7 (2.16–5.36)	
Bone metastasis		
Yes	4.3 (2.09–6.63)	0.777
No	3.7 (2.53–5.00)	
Chemotherapy response		
CR+PR	7.0 (5.05–9.01)	0.001
SD+PD	2.8 (2.58–3.08)	
NE	2.0 (1.71–2.41)	
Histological grade		0.483
Adenocarcinoma, WD	2.7 (2.19–3.33)	
Adenocarcinoma, MD	4.1 (2.73–5.59)	
Adenocarcinoma, PD	4.1 (2.25–5.94)	
Signet ring cell carcinoma	2.7 (2.45–3.07)	
Unknown	2.8 (2.06–3.60)	
ECOG PS		
2	4.4 (3.79–5.14)	0.001
3	1.9 (1.35–2.44)	
Albumin (g/dl)		
<3.5	2.8 (1.93–3.73)	0.158
≥3.5	4.1 (3.15–5.04)	
CRP (mg/dl)		
≤1.0	4.4 (3.52–5.41)	0.064
>1.0	3.0 (1.73–4.26)	
GPS		
0	4.6 (3.50–5.69)	0.006
≥1	2.8 (2.29–3.37)	

mPFS, median progression-free survival; CR, complete response; PR, partial response; SD, stable disease; PD, progressive disease; NE, not evaluable; WD, well-differentiated; MD, moderately differentiated; PD, poorly differentiated; ECOG PS, Eastern Cooperative Oncology Group performance status; CRP, C-reactive protein; GPS, Glasgow prognostic score.

**Table VII:** Overall survival and progression-free survival in advanced gastric cancer patients with poor performance status receiving single-agent chemotherapy (multivariate analysis).

Factors	Hazard ratio (95% CI)	P-value
Overall survival		
Chemotherapy regimen		0.016
Paclitaxel	1.40 (0.90–2.18)	0.129
Irinotecan	1.41 (0.77–2.58)	0.255
Capecitabine	5.00 (1.81–13.81)	0.002
No second-line chemotherapy	2.30 (1.48–3.57)	0.001
Bone metastasis	2.73 (1.22–6.09)	0.014
ECOG PS 3	38.10 (13.72–105.78)	0.001
GPS ≥1	1.88 (1.24–2.85)	0.003
Chemotherapy response		
SD+PD+NE	2.37 (1.39–4.05)	0.002
Progression-free survival		
Chemotherapy regimen		0.025
Paclitaxel	1.31 (0.85–2.03)	0.213
Irinotecan	1.12 (0.58–2.14)	0.728
Capecitabine	4.20 (1.64–10.85)	0.003
ECOG PS 3	5.86 (3.07–11.20)	0.001
Chemotherapy response		0.003
SD+PD+NE	2.41 (1.44–4.04)	0.001

ECOG PS, Eastern Cooperative Oncology Group performance status; GPS, Glasgow prognostic score; SD, stable disease; PD, progressive disease; NE, not evaluable. The reference value is TS-1 in chemotherapy regimen.
